# Stability of Cs_2_NaBiBr_6_ and Cs_2_NaBiCl_6_

**DOI:** 10.1021/acs.inorgchem.4c01299

**Published:** 2024-06-28

**Authors:** Minh N. Tran, Rafaella Saa Rodriguez, Joseph R. Geniesse, Kajini Sandrakumar, Iver J. Cleveland, Eray S. Aydil

**Affiliations:** Department of Chemical & Biomolecular Engineering, Tandon School of Engineering, New York University, New York, New York 11201, United States

## Abstract

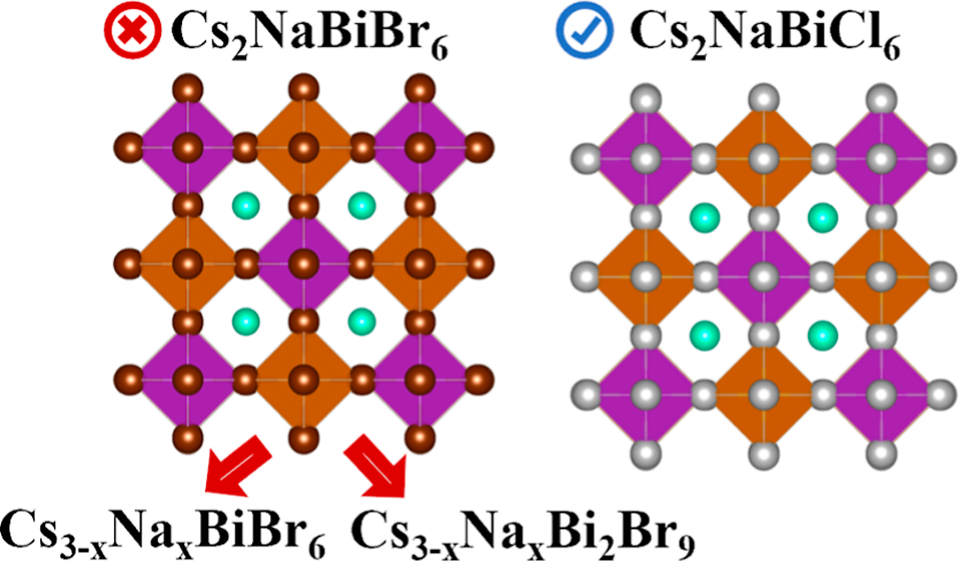

Bismuth-based halide perovskites are nontoxic alternatives to widely studied lead-based perovskites for optoelectronic applications. Here, we synthesized Cs_2_NaBiCl_6_ thin films and attempted to synthesize Cs_2_NaBiBr_6_ using physical vapor deposition. While Cs_2_NaBiCl_6_ forms a stable cubic structure with a 3.4 eV band gap and could be synthesized successfully, Cs_2_NaBiBr_6_ does not form and is unstable with respect to dissociation into Cs_3–*x*_Na_*x*_Bi_2_Br_9_ and Cs_3–*x*_Na_*x*_BiBr_6_. Furthermore, the close X-ray diffraction patterns of Cs_3–*x*_Na_*x*_Bi_2_Br_9_ and Cs_2_NaBiBr_6_ raise doubts about the previous reports of the latter’s formation based on X-ray diffraction alone.

## Abstract 2

We synthesized Cs_2_NaBiCl_6_ thin films and attempted to synthesize Cs_2_NaBiBr_6_ using physical vapor deposition. Cs_2_NaBiCl_6_ crystallizes with a stable cubic structure and a bandgap of 3.4 eV. Raman spectroscopy and X-ray diffraction reveal that Cs_2_NaBiBr_6_ does not form and is unstable with respect to dissociation into Cs_3–*x*_Na_*x*_Bi_2_Br_9_ and Cs_3–*x*_Na_*x*_BiBr_6_. Diffraction patterns of Cs_3–*x*_Na_*x*_Bi_2_Br_9_ and Cs_2_NaBiBr_6_ are close, and the former may be mistaken for the latter, raising doubts about the previous reports of Cs_2_NaBiBr_6_ formation.

## Introduction

**Figure 1:**
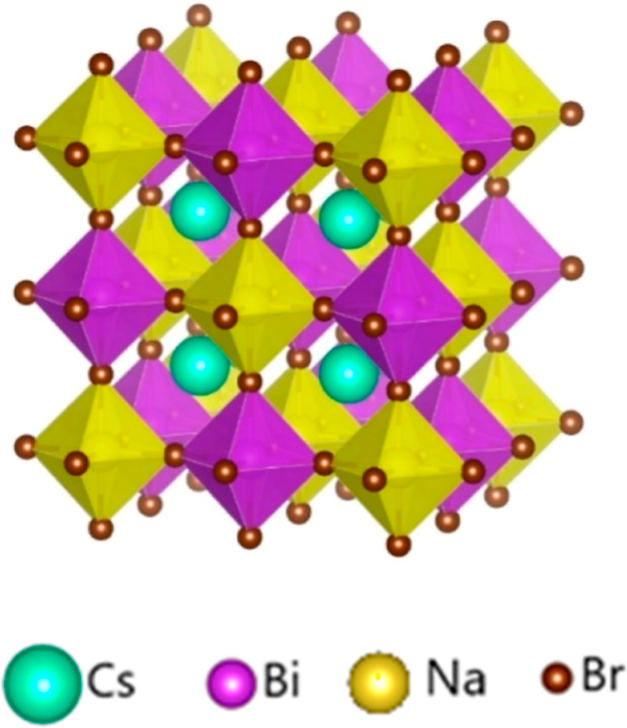
Proposed cubic structure of Cs_2_NaBiBr_6_ (#225, *Fm*3̅*m*). Images are produced by VESTA software.^21^

Inorganic halide perovskites have attracted significant attention for solar cells and other optoelectronic applications because of their stability compared to their hybrid organic–inorganic counterparts, tunable band gaps, strong absorption, and high-efficiency luminescence.^1−3^ While lead-based halide perovskites have shown excellent performance in these applications, lead toxicity is a major concern.^4,5^ The heterovalent substitution strategy to replace Pb^2+^ cations with monovalent and trivalent cations in alternating octahedra results in the double perovskite structure with the formula A_2_BB’X_6_ (Figure 1).^6^ In the search for lead-free perovskites, Cs_2_AgInX_6_ and Cs_2_AgBiX_6_ (X = Cl or Br) have emerged as potential candidates for applications in solar cell absorbers, photodetectors, and downconversion materials.^7−10^ However, the high costs of silver and indium pose challenges for large-scale production, motivating the exploration of low-cost alternatives such as Cs_2_NaBiCl_6_ and Cs_2_NaBiBr_6_.

**Figure 2:**
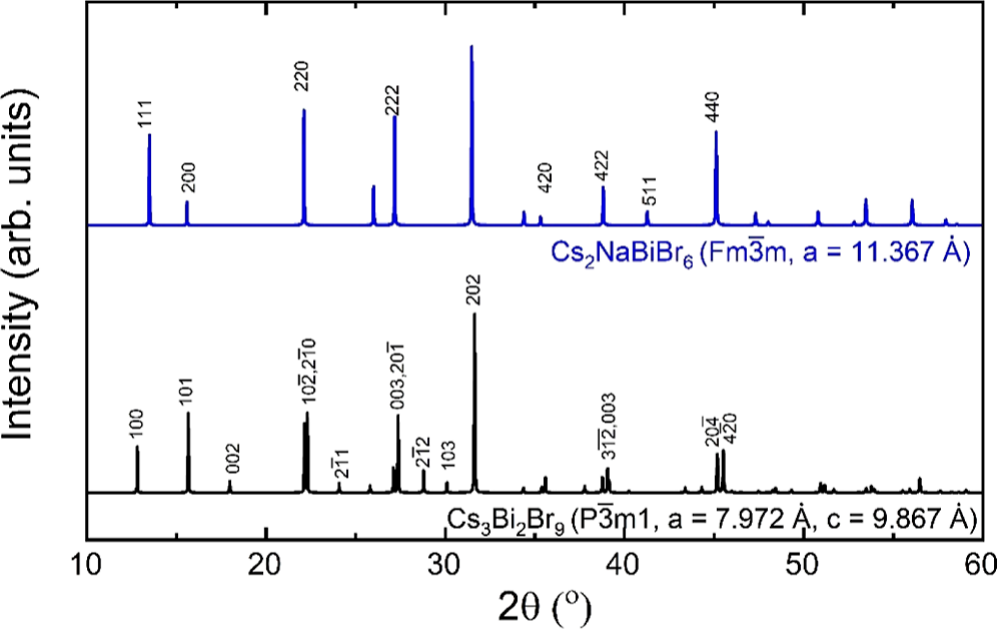
Simulated (using VESTA software)^21^ XRD pattern of Cs_2_NaBiBr_6_ (#225, *Fm*3̅*m*) with *a* = 11.367 Å reported by Wang et al.^12^ The simulated XRD of Cs_3_Bi_2_Br_9_ (#164, *P*3̅*m1*, *a* = 7.972, *c* = 9.867 Å) is also included for comparison. The XRDs are shifted along the intensity axis to offset for clarity.

To our knowledge, only three papers reported synthesizing Cs_2_NaBiBr_6_ nanocrystals, but whether Cs_2_NaBiBr_6_ is stable remains unclear.^11−13^ Wang et al. and Lee et al. claimed the formation of phase-pure Cs_2_NaBiBr_6_ nanocrystals based on their X-ray diffraction (XRD) patterns, from which a cubic structure (#225, *Fm*3̅*m*) was deduced for Cs_2_NaBiBr_6_ (Figure 1).^11,12^ Wang et al. determined the lattice constant of Cs_2_NaBiBr_6_ nanocrystals from XRD data and obtained a value of 11.367 Å.^12^ With *a* = 11.367 Å, the simulated XRD pattern of Cs_2_NaBiBr_6_ is very close to the pattern of other bismuth-based perovskites, Cs_3_Bi_2_Br_9_ (Figure 2), making it difficult to conclusively determine the presence of Cs_2_NaBiBr_6_ by XRD, particularly if these other impurity phases can also be formed from the precursors in the synthesis. For instance, Cs_3_Bi_2_Br_9_ could form when attempting to synthesize Cs_2_NaBiBr_6_. Since nanocrystals give broad XRD peaks, the experimentally observed diffractions can be from the targeted compound, Cs_2_NaBiBr_6_, or the impurity phase Cs_3_Bi_2_Br_9_, thus raising the possibility of contamination from Cs_3_Bi_2_Br_9_ going undetected. Another open issue is the stability of Cs_2_NaBiBr_6_. Lamba et al.^13^ showed that the XRD pattern of Cs_2_NaBiBr_6_ nanocrystals they synthesized contained NaBr peaks, suggesting that perhaps Cs_2_NaBiBr_6_ is unstable and decomposes to Cs_3_Bi_2_Br_9_ and NaBr. Zhao et al. reported a positive formation enthalpy for Cs_2_NaBiBr_6_ from DFT calculations, suggesting that this compound may be thermodynamically unstable.^14^

In contrast to Cs_2_NaBiBr_6_, Cs_2_NaBiCl_6_ has been studied since 1972.^15^ Morrs and Robinson synthesized a Cs_2_NaBiCl_6_ single crystal and proposed a cubic structure (#225, *Fm*3̅*m*) with lattice parameter, *a* = 10.839 Å at room temperature. Since then, Cs_2_NaBiCl_6_ single crystals, powders, and nanocrystals have been synthesized, all with XRD patterns consistent with the crystal structure proposed by Morrs and Robinson.^11,12,16−20^ However, the XRD pattern of Cs_2_NaBiCl_6_ has some overlapping peaks with Cs_3_Bi_2_Cl_9_, requiring additional characterization techniques to differentiate the two compounds and ensure the phase purity of Cs_2_NaBiCl_6_. We synthesized Cs_2_NaBiCl_6_ and Cs_2_NaBiBr_6_ thin films via coevaporation of halide salts and examined their crystal structures and optical properties.

## Experimental Section

### Thin Film Deposition

Films were deposited using thermal evaporation of CsX, NaX, and BiX_3_ (X = Cl or Br) in a glove-boxed physical vapor deposition (PVD) system (Angstrom Engineering). For the synthesis of Cs_2_NaBiBr_6_, the three precursors CsBr (99.9%, Acros Organics), NaBr (99.5%, Thermo Scientific), and BiBr_3_ (99%, Alfa Aesar) were loaded into separate crucibles placed in three separate RADAK thermal evaporation sources and baked under vacuum overnight at 110, 100, and 60 °C, respectively. For the synthesis of Cs_2_NaBiCl_6_, the three precursors CsCl (99.9%, Sigma-Aldrich), NaCl (99.99%, Sigma-Aldrich), and BiCl_3_ (99.9%, Alfa Aesar) were loaded into separate crucibles and baked under vacuum overnight at 110, 100, and 60 °C, respectively. Quartz crucibles were used for BiBr_3_ and BiCl_3_, while alumina crucibles were used for all the other precursors. For both Cs_2_NaBiBr_6_ and Cs_2_NaBiCl_6_ depositions, all the precursors were simultaneously evaporated onto glass substrates (25 × 75 mm^2^) while maintaining the substrate temperatures at 30 °C throughout the deposition. Before loading them onto the glovebox substrate holder, the glass substrates were cleaned by sonicating them in a 1:1 solution by volume of isopropanol (99.5%, VWR) and acetone (ACS grade, VWR) for 30 min. The substrates were dried in an oven at 50 °C and cleaned by immersing them in an O_2_ plasma for 30 min in an Expanded Plasma Cleaner PDC-001-HP (Harrick Plasma). Quartz crystal microbalances (QCMs) were used to monitor the evaporation rate of each precursor throughout the depositions and keep them constant at a set point by using a closed feedback control loop that manipulated the evaporation temperature. The respective tooling factors for the evaporation sources were obtained by evaporating all precursors separately and calculating film thickness from interference fringes in their optical transmission. The tooling factors are the ratio of the deposition rate on the substrates to the deposition rate at the QCM position. The tooling factors of CsBr, NaBr, CsCl, and NaCl were 39.7% and those of BiBr_3_ and BiCl_3_ were 42.9%. During the deposition of Cs_2_NaBiBr_6_, CsBr, NaBr, and BiBr_3_ were coevaporated at 1.22, 0.41, and 1.0 Å/s, respectively. These rates gave a molar flux ratio of CsBr to NaBr to BiBr_3_ of 2:1:1. The three source temperatures were used as manipulated variables but were controlled at ∼560, ∼600, and ∼120 °C, respectively, to maintain constant evaporation rates. For the deposition of Cs_2_NaBiCl_6_, CsCl, NaCl, and BiCl_3_ were coevaporated at 1.27, 0.41, and 1.0 Å/s, respectively. These rates gave a molar flux ratio of CsCl to NaCl to BiCl_3_ of 2:1:1. The CsCl, NaCl, and BiCl_3_ source temperatures were controlled at ∼600, ∼630, and ∼150 °C to maintain these constant evaporation rates. The PVD system’s initial pressure was 10^–7^ Torr, which rose to ∼10^–6^ Torr during the depositions. For both Cs_2_NaBiBr_6_ and Cs_2_NaBiCl_6_, films were deposited for 30 min. The film thickness calculated from the precursors’ fluxes (i.e., the evaporation rates) is 510 ± 10 nm for Cs_2_NaBiCl_6_, within 2% error from the thickness calculated from interference fringes (Figure S1). The Cs_2_NaBiBr_6_ film is unstable and decomposes into multiple phases; for this reason, the interference fringe method was not used to determine its thickness.

### Thin Film Characterization

All films were characterized in the air under ambient conditions. After deposition, Cs_2_NaBiBr_6_ thin films were stored under nitrogen and taken out of the glovebox when they were ready to be characterized to prevent moisture-induced decomposition. For Cs_2_NaBiBr_6_ and Cs_2_NaBiCl_6_ films, XRD patterns were recorded using a Bruker D8 Discover General Area Detector Diffraction System (GADDS) equipped with a Cu–Kα source. Raman spectra were acquired using a Thermo Scientific DXR Raman microscope. The thin films were excited with a 785 nm laser, and Raman scattering in the range of 50–500 cm^–1^ was collected with a 50× Olympus objective, dispersed using a high resolution (2 cm^–1^) grating, and detected with a CCD detector. A Merlin field emission scanning electron microscope (Carl Zeiss, 5 kV, 110pA) was used to examine all films. Their average composition over an area of approximately 10 μm^2^ was determined using energy-dispersive X-ray spectroscopy (Oxford Instruments EDS) and vendor-provided sensitivity factors. Optical absorptions of the films were recorded using an Agilent Cary 5000 UV–vis–NIR spectrophotometer in the 200–2000 nm range. For all Cs_2_NaBiCl_6_ films, photoluminescence (PL) spectra were measured using a QuantaMaster-8075-21 (Horiba) spectrophotometer. Visible PL was excited at 360 nm (5 nm bandwidth) with double monochromator-filtered emission from an Xe-arc lamp and detected using a PMT detector.

## Results and Discussion

### Cs_2_NaBiBr_6_

**Figure 3:**
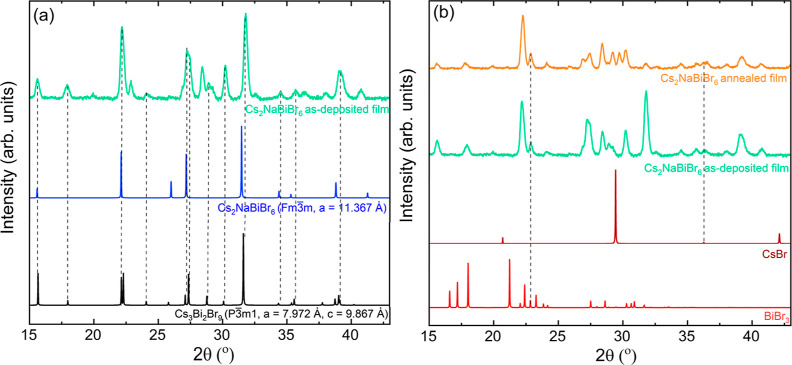
(a) XRD patterns from a film deposited by coevaporating CsBr, NaBr, and BiBr_3_ in the flux ratios of 2:1:1 to form Cs_2_NaBiBr_6_, simulated XRD of cubic Cs_2_NaBiBr_6_ (#225, *Fm*3̅*m*) with *a* = 11.367 Å as reported by Wang et al.,^12^ and simulated XRD pattern of Cs_3_Bi_2_Br_9_ (#164, *P*3̅*m1*). (b) XRD pattern of a film deposited by coevaporating CsBr, NaBr, and BiBr_3_ in the flux ratios of 2:1:1 to form a Cs_2_NaBiBr_6_ thin film and the same film after annealing at 300 °C for 1 h. The XRD patterns of CsBr and BiBr_3_ are also shown for comparison. The XRD patterns are shifted along the intensity axis to offset for clarity.

The XRD pattern of the as-deposited Cs_2_NaBiBr_6_ thin film comprises broad and asymmetric peaks, suggesting mixed phases (Figure 3a). While some peaks match the expected XRD peaks for the Cs_2_NaBiBr_6_ cubic structure proposed by Wang et al.,^12^ all these peaks can also be assigned to the layered perovskite Cs_3_Bi_2_Br_9_, as shown by the dotted lines in Figure 3a. Some peaks that do not match the reference Cs_2_NaBiBr_6_ structure can also be assigned to Cs_3_Bi_2_Br_9_ (e.g., 2θ_002_ = 17.98, 2θ_211_ = 24.08, 2θ_201̅_ = 27.37, 2θ_2̅12_ = 28.80, and 2θ_103_ = 30.10°). This suggests that the as-deposited Cs_2_NaBiBr_6_ film contains Cs_3_Bi_2_Br_9_, an impurity phase that was also observed by Lamba et al. in Cs_2_NaBiBr_6_ nanocrystals.^13^ This observation indicates that either the target cubic structure Cs_2_NaBiBr_6_ is unstable and decomposes to Cs_3_Bi_2_Br_9_ or the precursors do not react completely to form phase-pure Cs_2_NaBiBr_6_ during the deposition. Interestingly, the XRD peaks from the as-deposited Cs_2_NaBiBr_6_ film at high 2θ are slightly shifted compared to the corresponding expected locations in Cs_3_Bi_2_Br_9_ (e.g., 2θ_202_ = 31.81, 2θ_3̅10_ = 34.48, 2θ_2̅13_ = 35.66, and 2θ_300_ = 39.14°), indicating a small contraction in the Cs_3_Bi_2_Br_9_ unit cell. One likely hypothesis is that Na^+^ ions (116 ppm) substitute Cs^+^ ions (181 ppm) to form Cs_3–*x*_Na_*x*_Bi_2_Br_9_, which has a smaller lattice constant than Cs_3_Bi_2_Br_9_. The XRD peaks of Cs_3–*x*_Na_*x*_Bi_2_Br_9_ will be shifted to higher 2θ values compared to the Cs_3_Bi_2_Br_9_ pattern, and the shifts would be more obvious at the high 2θ range, as we observed in Figure 3a. However, since the as-deposited Cs_2_NaBiBr_6_ film contains mixed phases, these presumed shifted XRD peaks can also belong to other impurity phases. XRD of the as-deposited Cs_2_NaBiBr_6_ film also exhibits additional peaks at 19.94, 22.89, 28.42, 36.37, 38.07, and 40.77° that do not belong to Cs_3_Bi_2_Br_9_. The peak at 36.30° can be assigned to the (111) diffraction of the precursor CsBr, which supports the hypothesis that Na^+^ ions substitute Cs^+^ ions to form Cs_3–*x*_Na_*x*_Bi_2_Br_9_ (Figure 3b): such Na substitution for Cs and the formation of a phase with a 3:2 Cs/Bi ratio instead of a 2:1 Cs/Bi ratio would leave excess CsBr. The peak at 22.89° can be assigned to BiBr_3_, suggesting that the as-deposited film may also contain Bi-deficient phases. One common deficient Bi phase is Cs_3_BiBr_6_. Cs_3_BiBr_6_ XRD patterns contain many peaks that can overlap due to peak broadening (Figure S2). Thus, comparing the XRD pattern of Cs_3_BiBr_6_ and the as-deposited Cs_2_NaBiBr_6_ film is challenging, especially when Na^+^ ions can also substitute Cs^+^ ions (181 ppm) to form Cs_3–*x*_Na_*x*_BiBr_6_. Interestingly, the cubic structure Cs_2_NaBiBr_6_ (#225, *Fm*3̅*m*) and monoclinic (#15, *C12/c1*) (Cs_1–*x*_Na_*x*_)_3_BiBr_6_ have the same elemental composition when *x* = 1/3 (20 Cs, 10 Na, 10 Bi, 60% Br).^22^ Since the Na^+^ ion radius (116 ppm) is close to the Ag^+^ ion (129 ppm), we hypothesize that Cs_2_NaBiBr_6_ will crystallize in the same cubic structure as Cs_2_AgBiBr_6_. However, Na^+^ ions may also occupy Cs^+^ ions in the crystal structure and form the so-called 0-D monoclinic (Cs_1–*x*_Na_*x*_)_3_BiBr_6_ structure. It is called 0-D because the structure comprises isolated BiBr_6_^3–^ octahedra.

In summary, XRD shows that the as-deposited film does not form the cubic Cs_2_NaBiBr_6_ and contains Cs_3_Bi_2_Br_9_, Cs_3–*x*_Na_*x*_Bi_2_Br_9_, and possibly Cs_3_BiBr_6_, and (Cs_1–*x*_Na_*x*_)_3_BiBr_6._ The precursors are also present in the XRD pattern, raising questions about the stability of Cs_2_NaBiBr_6_ and whether it decomposes to the ternary phases, CsBr and BiBr_3_. Since the XRD pattern of as-deposited Cs_2_NaBiBr_6_ can be traced to multiple impurity phases, there are multiple decomposition pathways that Cs_2_NaBiBr_6_ can undergo, with each route leading to different products. For example, some decomposition pathways may be
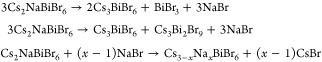


**Figure 4:**
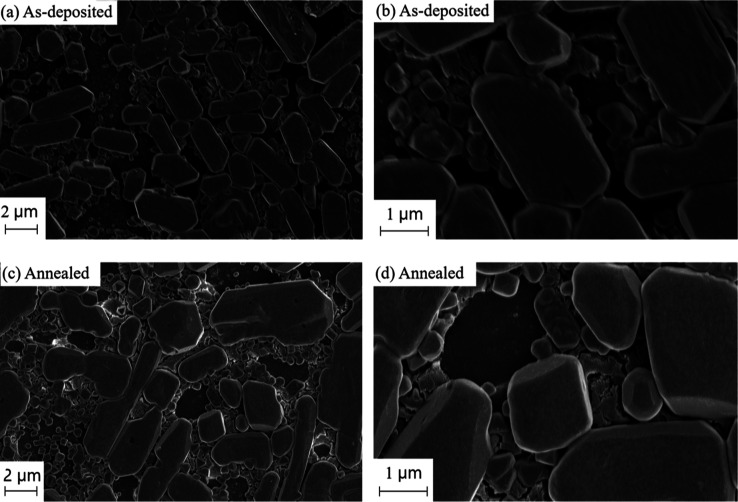
SEM images of a film deposited by coevaporating CsBr, NaBr, and BiBr_3_ in the flux ratios of 2:1:1 to form Cs_2_NaBiBr_6_ (a,b) and the same film annealed at 300 °C for 1 h (c,d).

Due to the complicated XRD pattern of the Cs_3_BiBr_6_ crystal structure and possibilities of peak overlapping from the peak broadening, it is challenging to quantify the relative amounts of each impurity phase from the thin film’s XRD, especially when the as-deposited thin film is unstable and may continue decomposing at the time of the measurement, and the impurity phases can also react with each other to form a new product. There is also the added complication of texturing. For instance, the measured XRD does not present the most intense peaks in the CsBr or BiBr_3_ powder diffraction pattern. When the as-deposited Cs_2_NaBiBr_6_ film decomposes, the impurities form large crystals with preferred orientations, as shown in the scanning electron microscopy (SEM) images (Figure 4), and we do not expect to observe all planes and XRD peaks from BiBr_3_ or CsBr. We use Raman spectroscopy and SEM to confirm the presence of Cs_3_BiBr_6_ and (Cs_1–*x*_Na_*x*_)_3_BiBr_6_, which is discussed in the next section.

The XRD of the as-deposited film raises two possibilities: either Cs_2_NaBiBr_6_ is unstable or the precursors do not react completely to form phase-pure Cs_2_NaBiBr_6_. The as-deposited film was annealed at 300 °C for 1 h under nitrogen to facilitate the reaction between precursors and distinguish between these two possibilities. The annealed film shows the same XRD peaks as the as-deposited Cs_2_NaBiBr_6_ films (Figure 3b), albeit with some changes in peak intensities, suggesting that the same compounds in the as-deposited film are present as in the annealed films but in different amounts. Thus, we conclude that cubic Cs_2_NaBiBr_6_ is unstable, and if it ever forms during annealing, it transforms into a mixture of compounds, which include Cs_3_Bi_2_Br_9_, Cs_3–*x*_Na_*x*_Bi_2_Br_9_, BiBr_3_, CsBr, and possibly Cs_3–*x*_Na_*x*_BiBr_6_ at room temperature.

**Figure 5:**
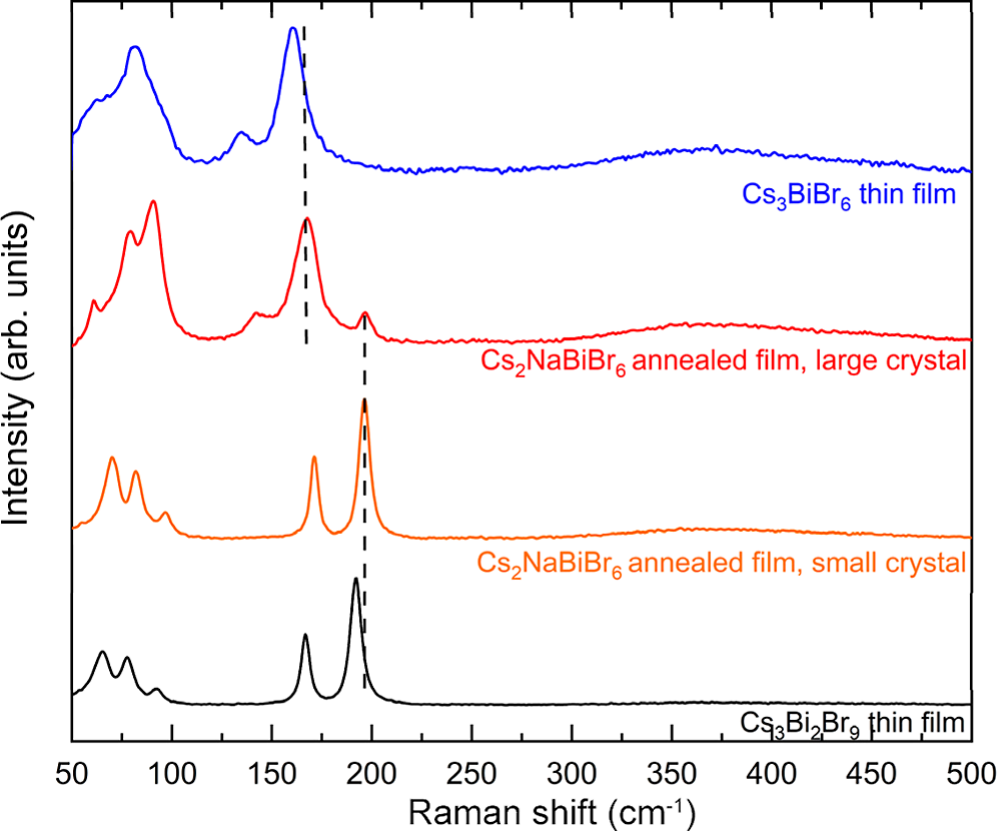
Raman spectra of the large and small crystal domains from a film deposited by coevaporating CsBr, NaBr, and BiBr_3_ in the flux ratios of 2:1:1 to form Cs_2_NaBiBr_6_ and annealed at 300 °C for 1 h. The Raman spectra of Cs_3_BiBr_6_ and Cs_3_Bi_2_Br_9_ thin films are also included for comparison. The spectra are shifted along the intensity axis to offset for clarity.

The SEM images of the as-deposited and annealed Cs_2_NaBiBr_6_ films are shown in Figure 4. Both films appear inhomogeneous and show a bimodal grain size distribution, a mixture of small (100–200 nm) and large (1–2 μm) grains. The average EDS composition of the large crystals is 24.2 Cs, 10.1 Na, 9.4 Bi, and 56.4% Br for the as-deposited film and 26.8 Cs, 6.5 Na, 9.6 Bi, and 57.2% Br for the annealed film. Both compositions (∼30 Cs and Na; ∼10 Bi and 60% Br) suggest the presence of a Bi-deficient and Cs-excess phase that matches layered A_3_BX_6_ perovskite or Cs_3–*x*_Na_*x*_BiBr_6_. The Raman spectrum of the large crystals recorded using a confocal microscope also shows peaks at 167 and 142 cm^–1^, which are signature peaks associated with the isolated [BiBr_6_]^3–^ octahedra in Cs_3–*x*_Na_*x*_BiBr_6_ (Figure 5). Those two peaks are shifted to higher wavenumbers compared to Cs_3_BiBr_6_ Raman peaks at 161 and 135 cm^–1^,^22^ which agrees with the hypothesis that Na^+^ ions substitute Cs^+^ ions in the crystal structure and form Cs_3–*x*_Na_*x*_BiBr_6_, contracting the lattice. The Raman peaks shift to higher wavenumbers when the structure is compressed, consistent with the smaller radius of Na^+^ ions substituting for the larger Cs^+^ ions. Quantitative determination of the content of Na substitution (x in Cs_3–*x*_Na_*x*_BiBr_6_ and Cs_3–*x*_Na_*x*_Bi_2_Br_9_) may be possible from XRD peak shifts but requires careful calibration of the XRD peak positions as a function of controlled Na addition into neat Cs_3_BiBr_6_ and Cs_3_Bi_2_Br_9_ films first. Such studies may be undertaken in the future, though it would still be challenging to monitor those changes directly from the unstable Cs_2_NaBiBr_6_ thin film as multiple decomposition pathways take place simultaneously and at different rates.

EDS compositions of the domains containing small crystals vary significantly from each other. Table S1 shows the composition of three areas comprising the majority of small crystals from the annealed film. The large composition variation in EDS analysis suggests that the small crystals are a mixture of phases. The Raman spectrum of domains with small crystals shows signature peaks at 196 and 171 cm^–1^, which are associated with two characteristic A_1g_ and E_g_ normal modes of Bi–Br vibration in the corner-sharing [BiBr_6_]^3–^ octahedra in Cs_3_Bi_2_Br_9_ (Figure 5). Those two peaks are also shifted to higher wavenumbers compared to Cs_3_Bi_2_Br_9_ Raman peaks at 192 and 167 cm^–1^, suggesting that Na^+^ ions substitute Cs^+^ ions to form Cs_3–*x*_Na_*x*_Bi_2_Br_9_. Additionally, the Raman spectrum of the Cs_3–*x*_Na_*x*_BiBr_6_ large crystal also has a small peak at 196 cm^–1^, so this large crystal can be formed from the domain that is made of Cs_3–*x*_Na_*x*_Bi_2_Br_9_ via the reaction
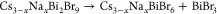


Consistent with this reaction and BiBr_3_ peaks in the XRD pattern, we also observe long rod-shaped crystals of BiBr_3_ in the film. The EDS composition of the region with these nanorods shows 17.8 Bi, 68.7 Br, 3.6 Cs, and 9.9% Na (Figure S3). A small amount of Cs is detected in the neighboring regions, and higher amounts of sodium are detected because of its presence in the glass substrate.

**Figure 6:**
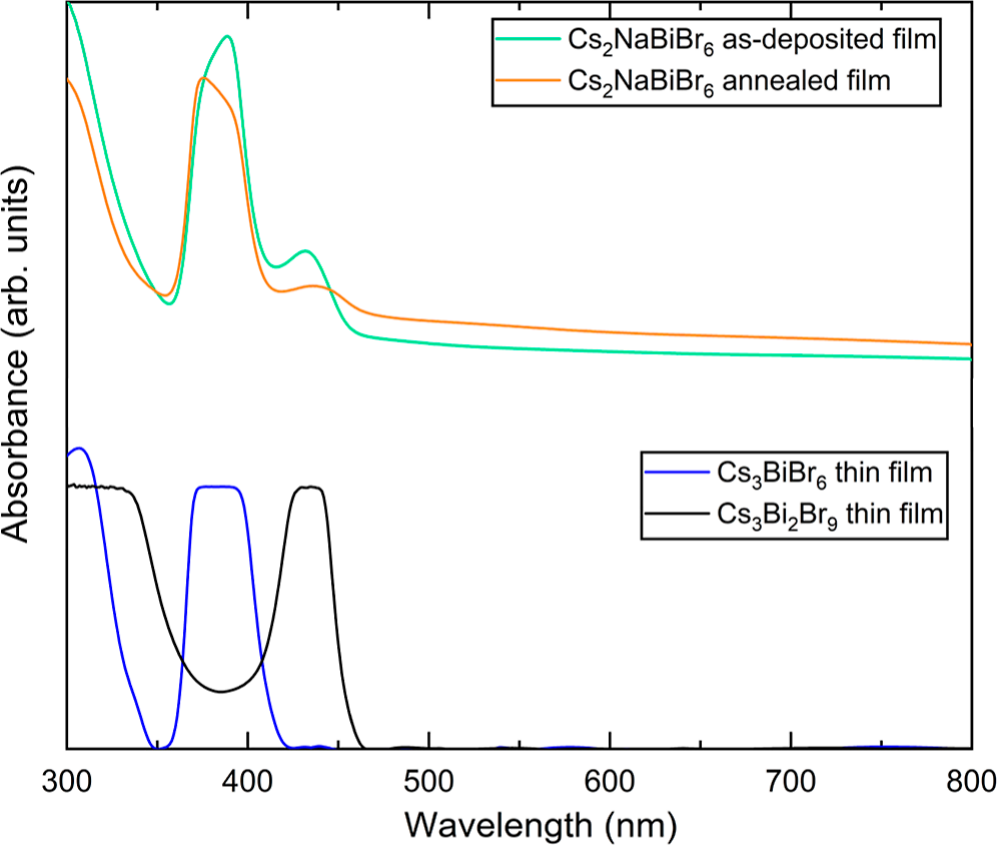
Optical absorption of a film deposited by coevaporating CsBr, NaBr, and BiBr_3_ in the flux ratios of 2:1:1 to form Cs_2_NaBiBr_6_ as-deposited and after annealing at 300 °C for 1 h. Optical absorption from Cs_3_BiBr_6_ and Cs_3_Bi_2_Br_9_ thin films is also shown for comparison. The spectra are shifted along the absorbance axis to offset for clarity. Thin film interference fringes in the raw absorption data were modeled and subtracted for the Cs_3_BiBr_6_ and Cs_3_Bi_2_Br_9_ absorption data.

The as-deposited and annealed films’ optical absorption spectra (Figure 6) show two peaks at 389 and 433 nm, close to the absorption peaks at 383 nm from Cs_3_BiBr_6_ and 435 nm from Cs_3_Bi_2_Br_9_, which corresponds to a localized exciton on isolated and corner-sharing [BiBr_6_]^3–^ octahedra, respectively.^22^ Thus, we associate the absorption peaks at 389 and 433 nm with the localized exciton on isolated [BiBr_6_]^3–^ octahedra in Cs_3–*x*_Na_*x*_BiBr_6_ and on corner-sharing [BiBr_6_]^3–^ octahedra in Cs_3–*x*_Na_*x*_Bi_2_Br_9_, respectively.

Thus, all our data suggests that Cs_2_NaBiBr_6_ is thermodynamically unstable, which agrees with the positive formation enthalpy of Cs_2_NaBiBr_6_ reported by Zhao et al.^14^ Previous works by Wang et al. and Lee et al. that claimed to have synthesized Cs_2_NaBiBr_6_ nanocrystals used XRD to assert the Cs_2_NaBiBr_6_ formation,^11,12^ but as we show here, the XRD pattern of Cs_2_NaBiBr_6_ is too close to the Cs_3_Bi_2_Br_9_ and Cs_3–*x*_Na_*x*_Bi_2_Br_9_ impurity phases to make such conclusions. This raises doubts about the phase purity since Cs_2_NaBiBr_6_ and Cs_3_Bi_2_Br_9_ have similar XRD patterns.

One possible explanation is that Cs_2_NaBiBr_6_ can be stable in nanocrystalline form and becomes unstable in the bulk phase as a thin film in our work. However, Lamba et al. synthesized Cs_2_NaBiBr_6_ nanocrystals and also observed decomposition over time.^13^ The Cs_2_NaBiBr_6_ nanocrystal excitonic absorption peak broadened, and the XRD patterns show new impurity peaks after 1 week. These observations suggest that the nanocrystals are also unstable and eventually decompose into other impurity phases. Both Lamba et al. and Zhao et al. report a positive formation enthalpy for Cs_2_NaBiBr_6_, suggesting that the material is thermodynamically unstable and will degrade spontaneously.^13,14^ These and our observations suggest that the instability of Cs_2_NaBiBr_6_ is unrelated to the crystal size or the synthesis method and is thermodynamic.

From the XRD and Raman analysis, we conclude that cubic Cs_2_NaBiBr_6_ (#225, *Fm*3̅*m*) does not form when stoichiometric amounts of CsBr, NaBr, and BiBr_3_ are evaporated in the 2:1:1 proportions. Instead of forming a 3D network of corner-shared octahedra, Na^+^ ions are more likely to substitute and compete with Cs^+^ ions in the crystal structure and form 0-D Cs_3–*x*_Na_*x*_BiBr_6_ and 2-D Cs_3–*x*_Na_*x*_Bi_2_Br_9_. The SEM images show the formation of multiple impurity phases, each with a different crystal size and shape. We obtained the compositional data of some representative crystals and assigned them to BiBr_3_ and Cs_3–*x*_Na_*x*_BiBr_6_. Other film domains consisting of smaller grains show significant composition variation, making it difficult to assign to a specific phase, rather than the fact that the film is highly unstable and transforms into multiple impurities.

### Cs_2_NaBiCl_6_

**Figure 7:**
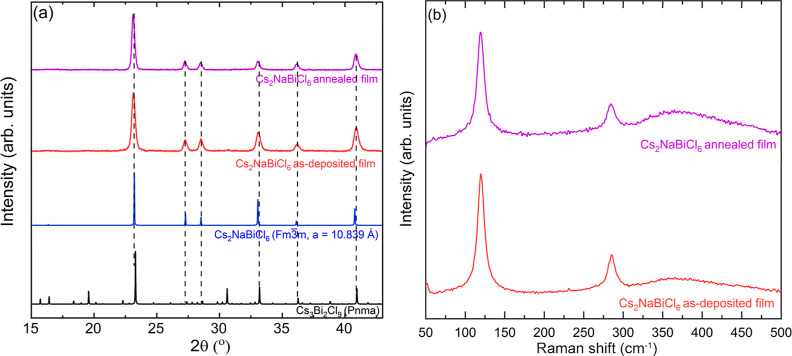
(a) XRD and (b) Raman spectra of as-deposited and annealed (300 °C, 1 h) Cs_2_NaBiCl_6_ films. The XRD patterns of Cs_2_NaBiCl_6_ and Cs_3_Bi_2_Cl_9_ are also included for comparison. The Raman spectra and XRD patterns are shifted along the intensity axis to offset data for clarity.

The XRD pattern of the as-deposited Cs_2_NaBiCl_6_ film, shown in Figure 7, comprises sharp and symmetric peaks that match the spectrum simulated using the cubic structure (#225, *Fm*3̅*m*) proposed by Morrs and Robinson.^15^ We do not observe any XRD peaks from unreacted precursors or impurity phases such as Cs_3_Bi_2_Cl_9_. Annealing Cs_2_NaBiCl_6_, the film at 300 °C for 1 h under nitrogen does not affect the XRD pattern. XRD remains unchanged after the Cs_2_NaBiCl_6_ annealed film is stored under ambient conditions for 3 days, suggesting that the cubic Cs_2_NaBiCl_6_ film is stable. However, since the XRD pattern of Cs_2_NaBiCl_6_ is very close to that of Cs_3_Bi_2_Cl_9_ and the film can be textured, we use Raman spectroscopy to verify the formation of phase-pure Cs_2_NaBiCl_6_.

**Figure 8:**
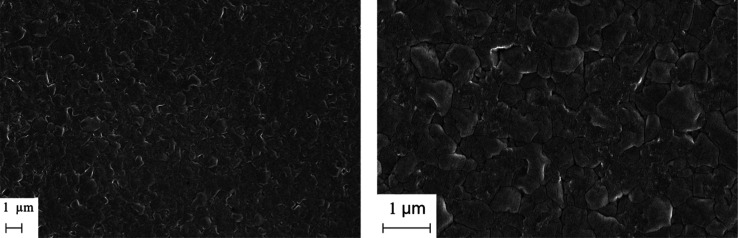
SEM images of an as-deposited Cs_2_NaBiCl_6_ thin film.

The Raman spectra of both the as-deposited and annealed Cs_2_NaBiCl_6_ thin film have two peaks at 120 and 286 cm^–1^, which agrees with the reported spectra for Cs_2_NaBiCl_6_ powders (Figure 8).^17,18,23^ Pelle et al. and Smit et al. also reported two additional Raman peaks of Cs_2_NaBiCl_6_ at 44 and 223 cm^–1^ at room temperature.^17,18^ We do not observe the peak at 223 cm^–1^ because of its low intensity, while the peak at 44 cm^–1^ is outside the detector range. Two peaks at 120 and 286 cm^–1^ are assigned to the T_2g_ and A_1g_ vibrational modes of the [BiCl_6_]^3–^ octahedra, respectively.^17,18^ Additionally, the Raman spectrum of the Cs_2_NaBiCl_6_ thin film is quite different from the reported spectrum of Cs_3_Bi_2_Cl_9_, which exhibits two peaks at 210 and 257 cm^–1^.^24,25^ This difference confirms the formation of the phase-pure Cs_2_NaBiCl_6_ structure in the vapor-deposited films. The SEM images of the as-deposited Cs_2_NaBiCl_6_ thin film also show a homogeneous, uniform film with an EDS composition of 20.6 Cs, 15.4 Na, 10.4 Bi, and 53.6% Cl, which is close to the expected theoretical values of 20 Cs, 10 Na, 10 Bi, and 60% Cl. The higher concentration of Na can be due to the presence of Na in the glass substrates.

**Figure 9:**
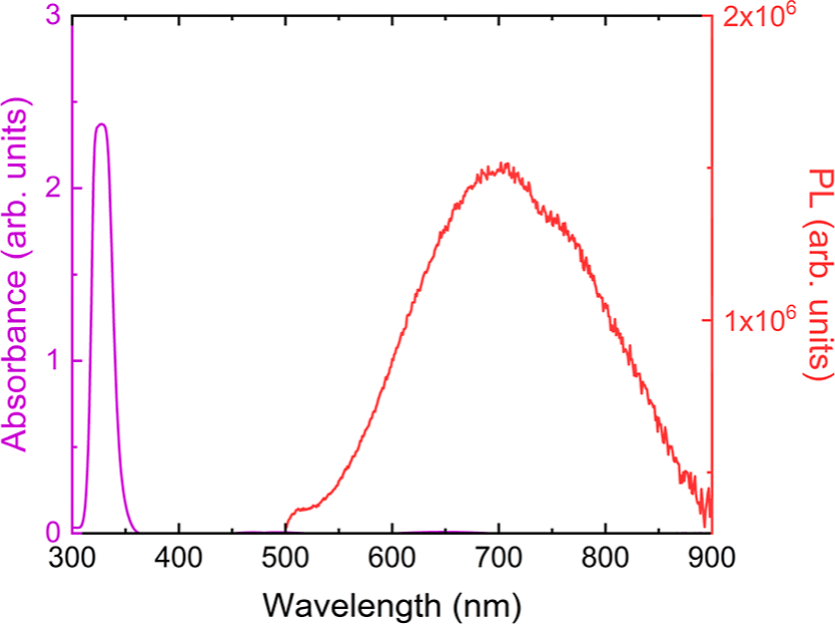
Absorption of and PL emission from a Cs_2_NaBiCl_6_ film annealed at 300 °C for 1 h. The film is excited with λ_ex_ = 360 nm. Thin film interference fringes were subtracted from the absorption data.

The Cs_2_NaBiCl_6_ thin film shows an absorption peak at 325 nm, which is also observed in Cs_2_NaBiCl_6_ bulk and nanocrystals (Figure 9).^11,20,23^ From our previous publications on bismuth-based perovskites, we assign this absorption peak to a localized exciton on the corner-shared [BiCl_6_]^3–^ octahedra in Cs_2_NaBiCl_6_. We extract the band gap of 3.4 eV (∼365 nm) from the absorption of the Cs_2_NaBiCl_6_ thin film, which is close to the calculated range of 3.4 to 3.7 eV for Cs_2_NaBiCl_6_.^12,20,23,26^ When excited at 360 nm, the Cs_2_NaBiCl_6_ thin film exhibits a weak and broad emission centered at 700 nm (fwhm = 200 nm). This broad emission is also observed in single crystals and powders.^16,19,23^ Due to the large Stokes shift, this broad emission at 700 nm is likely due to defects, though emission from self-trapped excitons has been suggested in similar materials.^27,28^

## Conclusions

We coevaporated CsX, BiX_3_, and NaX (X = Cl, Br) stoichiometrically to yield Cs_2_NaBiBr_6_ and Cs_2_NaBiCl_6_ thin films. The Cs_2_NaBiBr_6_ film appears unstable and forms a mixture of phases rather than the hypothesized cubic structure for double perovskites. The XRD pattern of the hypothesized Cs_2_NaBiBr_6_ cubic structure is very close to the pattern of Cs_3_Bi_2_Br_9_, which makes it difficult to differentiate the two phases using XRD analysis alone. We use a combination of XRD, Raman, SEM, and EDS to show that Na^+^ ions likely substitute Cs^+^ ions in the crystal structure to form Cs_3–*x*_Na_*x*_Bi_2_Br_9_ and Cs_3–*x*_Na_*x*_BiBr_6_. We also successfully deposited a Cs_2_NaBiCl_6_ thin film, which attains a cubic crystal structure with a band gap of 3.4 eV. The film is stable and remains phase-pure under ambient conditions for days.

## Supporting Information Available

Experimental transmission curves and fits and XRD and EDS data including Cs_3_BiBr_6_ XRD patterns extracted from two proposed structures, EDS composition of the domain containing small cubic crystals in the Cs_2_NaBiBr_6_ annealed film, and image of the long rod-shaped crystals in the Cs_2_NaBiBr_6_ thin film (PDF)

## Abbreviations

XRD: X-ray diffraction
SEM: scanning electron microscopy
EDS: energy-dispersive X-ray spectroscopy
