# Supplementary material for: Stability of Cs2NaBiBr6 and Cs2NaBiCl6
Source: Inorg Chem. 2024 Jun 28;63(28):12818–25. doi: 10.1021/acs.inorgchem.4c01299 (PMC11256743; doi:10.1021/acs.inorgchem.4c01299)
Supplement: Supplementary file 1 — ic4c01299_si_001.pdf [file ic4c01299_si_001.pdf]

Supporting Information

Stability of $Cs_2NaBiBr_6$ and $Cs_2NaBiCl_6$

*Minh N. Tran, Rafa S. Rodriguez, Joseph R. Geniesse, Kajini Sandrakumar, Iver J. Cleveland, and Eray S. Aydil**

Department of Chemical & Biomolecular Engineering, Tandon School of Engineering, New York University, New York, NY 11201, United States

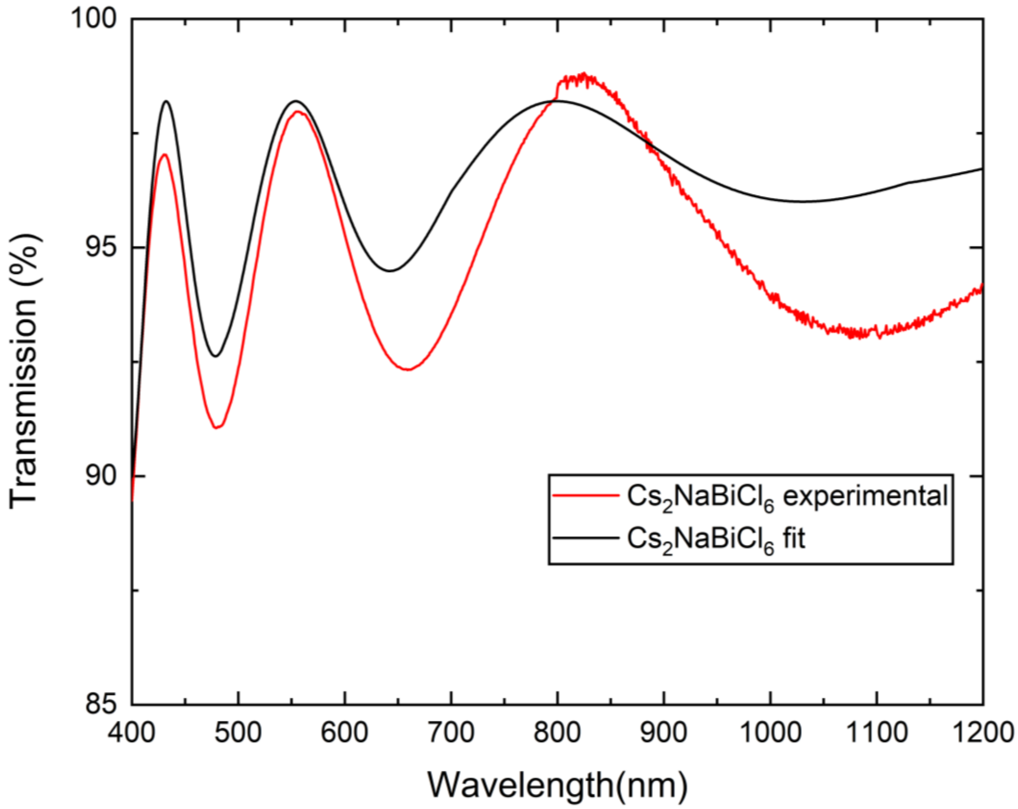

Figure S1 Experimental transmission curves and fits using film thicknesses of 500nm for $Cs_2NaBiCl_6$. The refractive indices are extracted from Shah *et al.*[1]

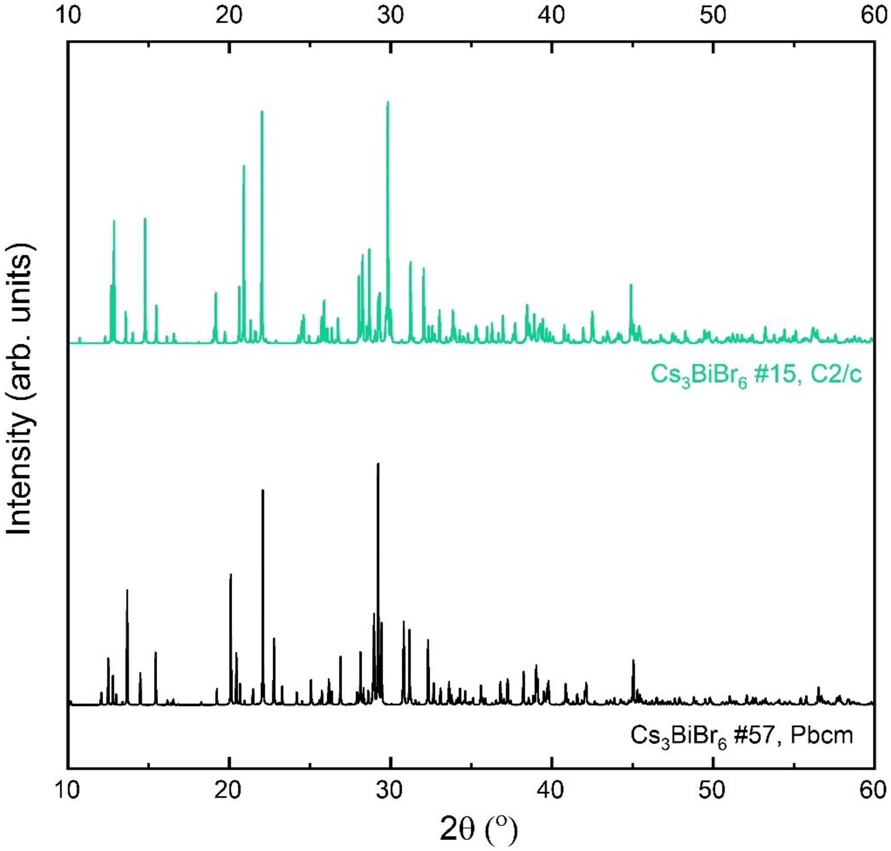

Figure S2. Cs_3BiBr_6 XRD patterns extracted from two proposed structures by Yang *et al.* (#15, C2/c,) and Tang *et al.* (#57, *Pbcm*).[2,3]

Table 1. EDS composition of the domain containing small cubic crystals in $Cs_2NaBiBr_6$ annealed film. The target composition of $Cs_2NaBiBr_6$ is 20% Cs, 10% Na, 10% Bi, and 60% Br.

**% Cs**	**% Na**	**% Bi**	**% Br**
18.6	18.1	11.9	51.4
24.3	10.1	9.6	55.9
10.1	51.7	6.8	31.5

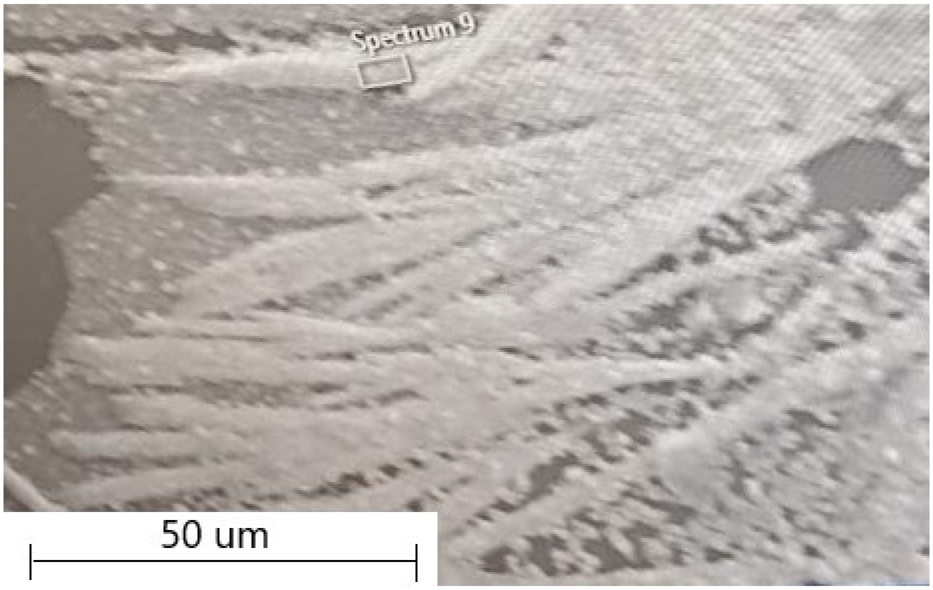

Figure S3. Long-rod shaped crystals in the $Cs_2NaBiBr_6$ thin film with the EDS composition of 17.8% Bi, 68.7% Br, 3.6% Cs and 9.9% Na. The EDS composition suggests the long rod consists mainly of $BiBr_3$.

---

[1] Zuhair Abbas Shah, S.; Niaz, S.; Nasir, T.; Sifuna, J. First Principles Insight into Band Gap Tuning in Bismuth Based Double Perovskites $X_2NaBiCl_6$ (X=Cs, Rb, K) for Enhanced Optoelectronic and Thermoelectric Properties. *Results in Chemistry* **2023**, 5, 100828.

[2] Yang, H.; Cai, T.; Liu, E.; Hills-Kimball, K.; Gao, J.; Chen, O. Synthesis and Transformation of Zero-Dimensional Cs3BiX6 (X = Cl, Br) Perovskite-Analogue Nanocrystals. *Nano Research* **2019**, *13* (1), 282–291.

[3] Tang, Y.; Liang, M.; Chang, B.; Sun, H.; Zheng, K.; Pullerits, T.; Chi, Q. Lead-Free Double Halide Perovskite Cs3BiBr6 with Well-Defined Crystal Structure and High Thermal Stability for Optoelectronics. *Journal of Materials Chemistry C* **2019**, *7* (11), 3369–3374.
